# Long non-coding RNA DUXAP8 elevates RCN2 expression and facilitates cell malignant behaviors and angiogenesis in cervical cancer via sponging miR-1297

**DOI:** 10.1186/s13000-021-01145-9

**Published:** 2021-11-14

**Authors:** Jihui Gu, Yi Liu, Ting Qi, Weiwei Qian, Dongdong Hu, Wen Feng

**Affiliations:** Department of Gynecology, the First People’s Hospital of Lianyungang, No.6 Zhenhua East Road, Jiangsu 222000 Lianyungang, China

**Keywords:** Cervical cancer, DUXAP8, miR-1297, RCN2

## Abstract

**Background:**

Cervical cancer (CC) endangers women’s health in the world range. Accumulating studies have revealed the crucial regulatory role of long non-coding RNAs (lncRNAs) in multiple malignancies, including CC. Our study aimed to explore the role of lncRNA double homeobox A pseudogene 8 (DUXAP8) in cervical carcinogenesis.

**Methods:**

Gene expressions in CC were assessed by RT-qPCR. Function experiments and tube formation assays were performed to evaluate the role of DUXAP8 in CC cells. Subcellular fractionation and FISH assays were conducted to determine the subcellular location of DUXAP8. Luciferase reporter, RNA pull down and RIP assays were conducted to investigate the mechanism of DUXAP8.

**Results:**

DUXAP8 was notably upregulated in CC cells. Downregulation of DUXAP8 repressed cell malignant behaviors and angiogenesis in CC. Mechanically, DUXAP8 boosted the expression of reticulocalbin-2 (RCN2) through relieving the binding of miR-1297 to RCN2 3’-UTR. Moreover, miR-1297 inhibition and RCN2 overexpression could counteract the inhibitory effects of DUXAP8 knockdown on the malignant phenotypes of CC cells. Besides, enhanced RCN2 expression restored the tumor growth in vivo that was inhibited by DUXAP8 repression.

**Conclusions:**

DUXAP8 promotes malignant behaviors in CC cells via regulating miR-1297/RCN2 axis.

**Graphical Abstract:**

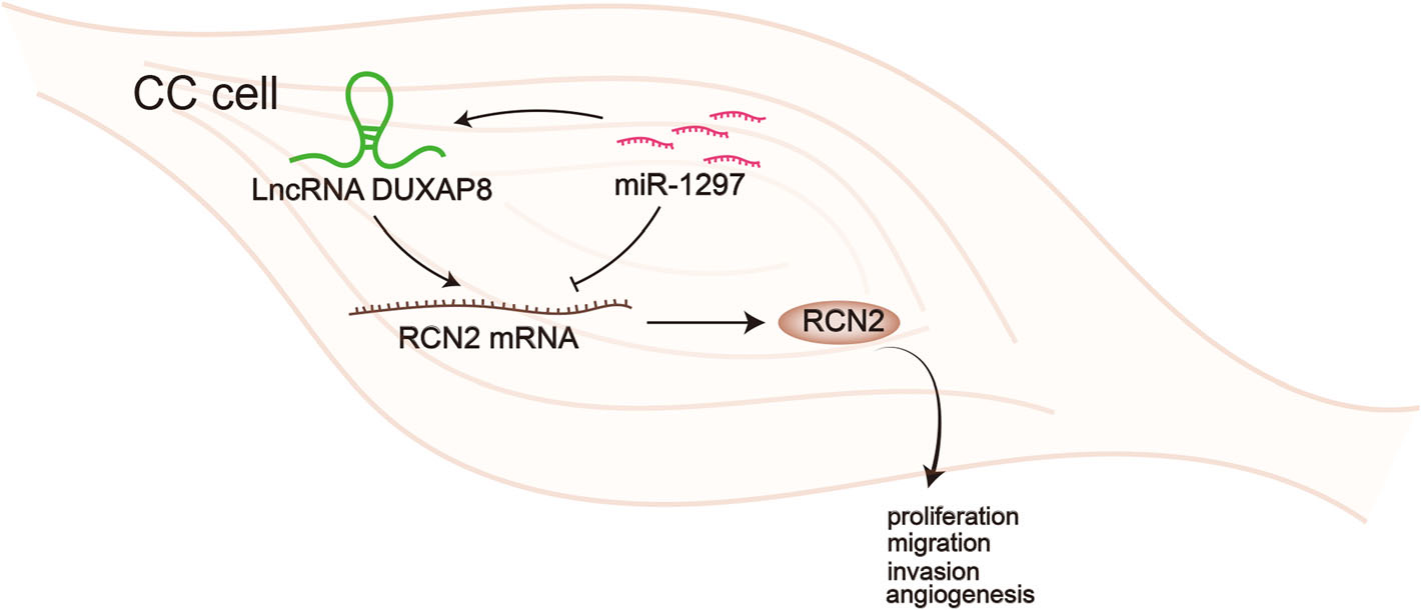

## Background

Cervical cancer (CC) remains one of the most common malignancies that undermines the health of females and a major health challenge in developing countries [1, 2]. Over the past decade, surgery operation, radiation oncology and chemotherapy have been developed to combat CC, yet the recurrence rate for patients remains 35 % [3]. In 2021, the estimated number of new cases of CC is 14,480 and the estimated death number of CC is 4290 in the United States [4]. Numerous reports have focused on gene therapy and explored the regulations between genes in various types of cancers. Herein, we aimed to elucidate the mechanism behind the carcinogenesis of CC.

Long non-coding RNAs (lncRNAs) are a group of non-coding RNAs (ncRNAs) over 200 nucleotides [5]. The dysregulation of lncRNAs in cellular processes has been discovered in an array of cancers, indicating the potential application of lncRNAs as diagnosis biomarkers and therapeutic targets for cancers [6] Meanwhile, findings on the molecular mechanisms and pathways of lncRNAs can be translated into clinical treatment for patients [7]. A great number of lncRNAs have been reported to play pivotal roles in a series of cellular biological processes, such as cell proliferation, differentiation, apoptosis, migration and epithelial mesenchymal transition (EMT) [8, 9]. For instance, DUXAP8 knockdown suppresses cell proliferation and facilitates cell apoptosis in pancreatic cancer [10]. Moreover, lncRNAs have been discovered to function as a class of competing endogenous RNAs (ceRNAs) to post-transcriptionally regulate gene expressions. Under ceRNA networks, lncRNAs could restore the expression of specific messenger RNAs (mRNAs) via sponging miRNAs through physical interaction [11]. For example, DUXAP8 promotes the progression of hepatocellular carcinoma by sponging miR-422a and enhancing PDK2 expression [12]. DUXAP8 serves as ceRNA for miR-577 to facilitate the invasion and migration of colorectal cancer cells via regulating RAB14 [13]. The similar mechanism of lncRNAs in CC has also been uncovered. For instance, silencing LINC01305 inhibited the PI3K/Akt signaling pathway in CC via targeting TNXB [14]. CAR10 facilitates CC development by sponging miR-125b-5p and upregulating PDPK1. However, the performance of DUXAP8 in CC remains unexplored.

MiRNAs represent another kind of ncRNAs, which possess approximately 18–22 nucleotides in length [15]. To date, miRNAs have been extensively reported to participate in the pathogenesis of various cancers [16]. More importantly, previous studies have highlighted the biological function of miRNAs in CC [17].

As a member of reticulocalbin (RCN) family, reticulocalbin-2 (RCN2) was revealed to exert oncogenic effects in several human malignancies. As an example, RCN2 was unveiled to be up-regulated in hepatocellular carcinoma (HCC) and its expression was positively correlated with tumor size in HCC patients [18]. However, the relationship between RCN2 and DUXAP8 has never been investigated in CC.

In this study, we aimed to investigate whether and how DUXAP8 regulates CC progression. The expression pattern of DUXAP8 was firstly determined in CC cells. Functions of DUXAP8 in CC progression were also detected. Importantly, we focused on the downstream molecular mechanism of DUXAP8 in CC. Collectively, we probed into the functions of DUXAP8 via specific miRNA/mRNA axis in CC.

## Methods

### Cell culture

Human CC cell lines (C4-1, caSki, HeLa and SiHa) and normal cervical cell line (Ect1/E6E7) were all procured from Shanghai Institute of Cell Biology (Shanghai, China). All cells were propagated in RPMI-1640 with 1 % antibiotics and 10 % FBS (Gibco, Grand Island, NY) under 5 % CO_2_ at 37℃.

### Total RNA extraction and real-time quantitative PCR (RT-qPCR)

Total RNAs were extracted from caSki and HeLa cell samples as instructed by the protocol of TRIzol reagent (Invitrogen, Carlsbad, CA). After that, RNA samples were subjected to PrimeScript™ RT Master Mix (TaKaRa, Shiga, Japan) for cDNA synthesis. SYBR Green PCR Kit (TaKaRa) was employed to perform qPCR, with GAPDH and U6 as the endogenous controls. Relative RNA expression was processed by 2^−ΔΔCt^ method.

### Plasmid transfection

Silencing of DUXAP8 and overexpression of RCN2 in caSki and HeLa cell samples were severally achieved by transfection with the duplex shRNAs (Genepharma, Shanghai, China) against DUXAP8 and pcDNA3.1/RCN2. The nonspecific shRNAs and empty pcDNA3.1 vector (Invitrogen) served as the negative control (NC). Besides, miR-1297 mimics/inhibitor and NC mimics/inhibitor were also procured from Genepharma. Cell transfection was performed for 48 h with Lipofectamine 2000 (Invitrogen).

### Colony formation assay

CC cells in the 6-well plates were seeded at 1 × 10^3^ cells/100µL for 14 days of incubation at 37℃ with 5 % CO_2_. 1 mL 0.1 % crystal violet solution was added to each well prior to counting colonies.

### EdU incorporation assay

CC cells were planted after transfection into 96-well plates with 8 × 10^3^ cells/well and then processed with the Cell-light™ EdU ApolloR567 in Vitro Imaging Kit (RiboBio, Guangzhou, China). Following fixation and permeabilization, samples were imaged by fluorescent microscope (Olympus Corp., Tokyo, Japan) after DAPI staining for cell nuclei.

### Annexin V staining assay

Transfected CC cell samples were harvested, rinsed in pre-chilled phosphate buffered saline (PBS), and then exposed to Annexin V FITC/PI detection kit (Invitrogen) as instructed by supplier for 15 min at 4℃. Finally, cell apoptosis rate was evaluated by analyzing Annexin V/PI-stained cells via flow cytometer (Beckman Coulter, Inc., Brea, CA).

### Transwell assay

Transwell assays were implemented using the transwell chamber (Corning Co, Corning, NY) with Matrigel (for invasion assay) or without Matrigel (for migration assay) as per the protocol. 5 × 10^4^ cell samples were added into the upper chamber, with complete medium added into the lower chamber. Invading or migrating cells were fixed in 4 % paraformaldehyde after 24 h, and then dyed with crystal violet solution. Stained cell samples were photographed under microscope and quantified.

### Tube formation assay

2.5 × 10^4^ human vascular endothelial cells (HUVECs) cultured in the conditional medium (CM) of caSki or HeLa cells were grown in 96-well plates with Matrigel at 37℃ for 6 h. The branches, representing the degree of angiogenesis in vitro, were observed and the number was quantitated under a light microscope (Nikon, Tokyo, Japan).

### Subcellular fractionation assay

Based on the protocol, the nuclear and cytoplasmic fractions were isolated from cell samples using PARIS™ Kit (Invitrogen). After treating with the cell fractionation and cell disruption buffer in sequence, DUXAP8 expression level in different fractions was determined by RT-qPCR, with GAPDH and U6 as indicated controls.

### RNA FISH analysis

For RNA FISH analysis, CC cell samples were fixed and air-dried for cultivation with DUXAP8 FISH probe (RiboBio) in the hybridization buffer. Hoechst solution was then added for the detection of cell nuclei, followed by the cells visualized with fluorescent microscope.

### Luciferase reporter assays

Recombinant luciferase reporter vectors DUXAP8 WT/Mut or RCN2 WT/Mut were acquired by inserting DUXAP8 or RCN2 fragments with the wild-type or mutated miR-1297 binding sites into the downstream of reporter vector pmirGLO (Promega, Madison, WI). Then the recombinant vectors were subjected to co-transfection with miR-1297 mimics or NC mimics for 48 h. After that, Luciferase Reporter Assay System (Promega) was employed for detecting the luciferase activity.

### RNA pull down assay

The wild type or mutant miR-1297 sequences covering DUXAP8 binding sites were synthesized and tagged with biotin into Biotin miR-1297 WT/Mut. The extracts from CC cell samples were mixed with the biotinylated RNA probes and streptavidin beads. The RNA in final pull-downs was assayed through RT-qPCR.

### RNA binding protein immunoprecipitation (RIP)

RIP assay was conducted by use of EZ-Magna RIP RNA Binding Protein Immunoprecipitation Kit (Millipore, Bedford, MA) as required by supplier. Cultured cell samples were lysed and then mixed with the beads-bound human anti-Ago2 antibody or normal control anti-IgG antibody (Millipore). After RNA purification, RT-qPCR analysis was conducted to verify the presence of indicated RNAs.

### In vivo xenograft experiments

Nine six-week-old BALB/c nude mice were acquired from Vital River Laboratories (Beijing, China). Xenograft tumor models were established via subcutaneously injecting caSki cells transfected with sh-NC, sh-DUXAP8#1 or sh-DUXAP8#1 + pcDNA3.1/RCN2 into above mice (randomly divided into three groups). The volume of neoplasms was monitored every 4 days. Four weeks later, mice were sacrificed and the weight of each tumor was measured. The animal study was approved by the Ethics Committee of the First People’s Hospital of Lianyungang.

### Statistical analysis

Data from independent bio-triplications were expressed as the mean ± SD. Differences between the different groups were processed through t test or one-way analysis of variance applying the PRISM 7.0 (GraphPad, San Diego, CA). Threshold of significance was set as *p*-value < 0.05.

## Results

### DUXAP8 is aberrantly highly expressed in CC and silencing DUXAP8 suppresses the malignant behaviors of CC cells

**Fig. 1:**
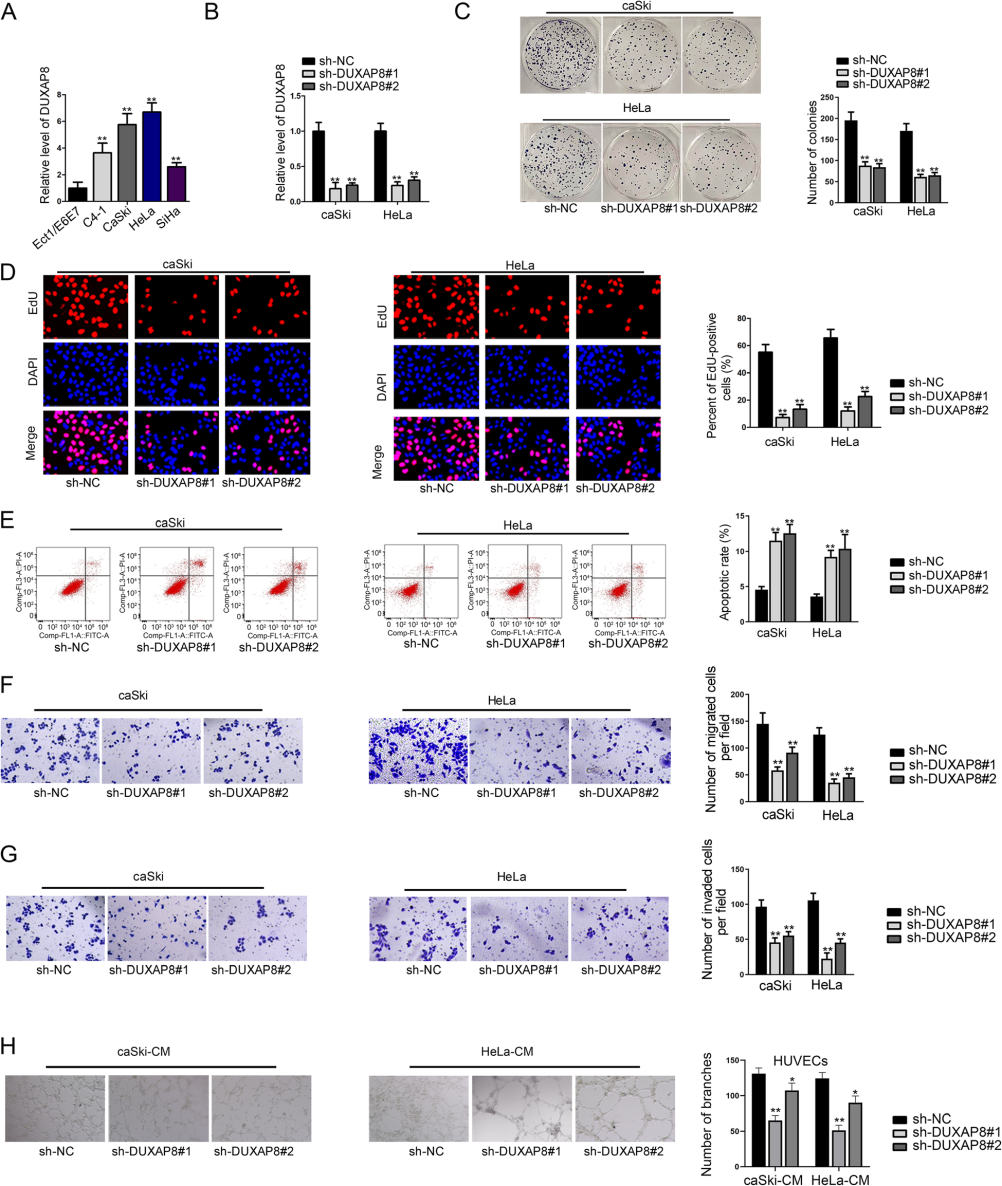
DUXAP8 is aberrantly highly expressed in CC and silencing DUXAP8 suppresses the malignant phenotypes of CC cells. **A**. The expression of DUXAP8 in CC cell lines and normal cervical cell line was detected by RT-qPCR analysis. **B**. The inhibitory efficiency of sh-DUXAP8#1/2 was detected by RT-qPCR. **C-D**. Colony formation assay and EdU assay were performed to test cell proliferation ability after transfecting sh-DUXAP8#1/2 into caSki and HeLa cells. **E**. Flow cytometry analysis was conducted to assess the apoptotic rate of cells with or without DUXAP8 inhibition. **F**. Transwell migration assay determined the migration of caSki and HeLa cells after silencing DUXAP8. **G**. The impact of DUXAP8 silence on the invasion of caSki and HeLa cells was evaluated by transwell invasion assay. **H**. Tube formation assays were performed to explore angiogenesis ability after silencing DUXAP8 in HUVECs co-cultured with the CM of indicated caSki and HeLa cells. **P < 0.01

RT-qPCR analysis was performed in CC cell lines and normal cervical epithelial cell line Ect1/E6E7 to explore the expression level of DUXAP8. Results manifested that DUXAP8 expression was markedly increased in CC cell lines, especially in caSki and HeLa cells (Fig. 1A), which suggested that DUXAP8 might be implicated in the progression of CC. To determine the role of DUXAP8 in CC, we adopted loss-of-function assays after ensuring the inhibitory efficiency of sh-DUXAP8#1/2 in caSki and HeLa cells (Fig. 1B). Colony formation and EdU assays indicated that silencing DUXAP8 evidently suppressed the proliferation of caSki and HeLa cells (Fig. 1C, D). In addition, flow cytometry analyzed that the rate of apoptotic cells was increased by silencing DUXAP8 (Fig. 1E), implying the promoting effect of DUXAP8 knockdown on CC cell apoptosis. More importantly, we found that the absence of DUXAP8 could impair the migration and invasion capacities of caSki and HeLa cells (Fig. 1F, G). Since angiogenesis is crucial for tumor growth and metastasis, we performed tube formation assay in HUVECs incubated with the conditional medium (CM) of caSki and HeLa cells. As a result, loss of DUXAP8 reduced the number of tube branches (Fig. 1H), indicating the repressive impact of downregulated DUXAP8 on angiogenesis. In sum, DUXAP8, upregulated in CC, strengthens the malignant phenotypes of CC cells.

### MiR-1297 binds to DUXAP8 and is negatively mediated by DUXAP8

**Fig. 2:**
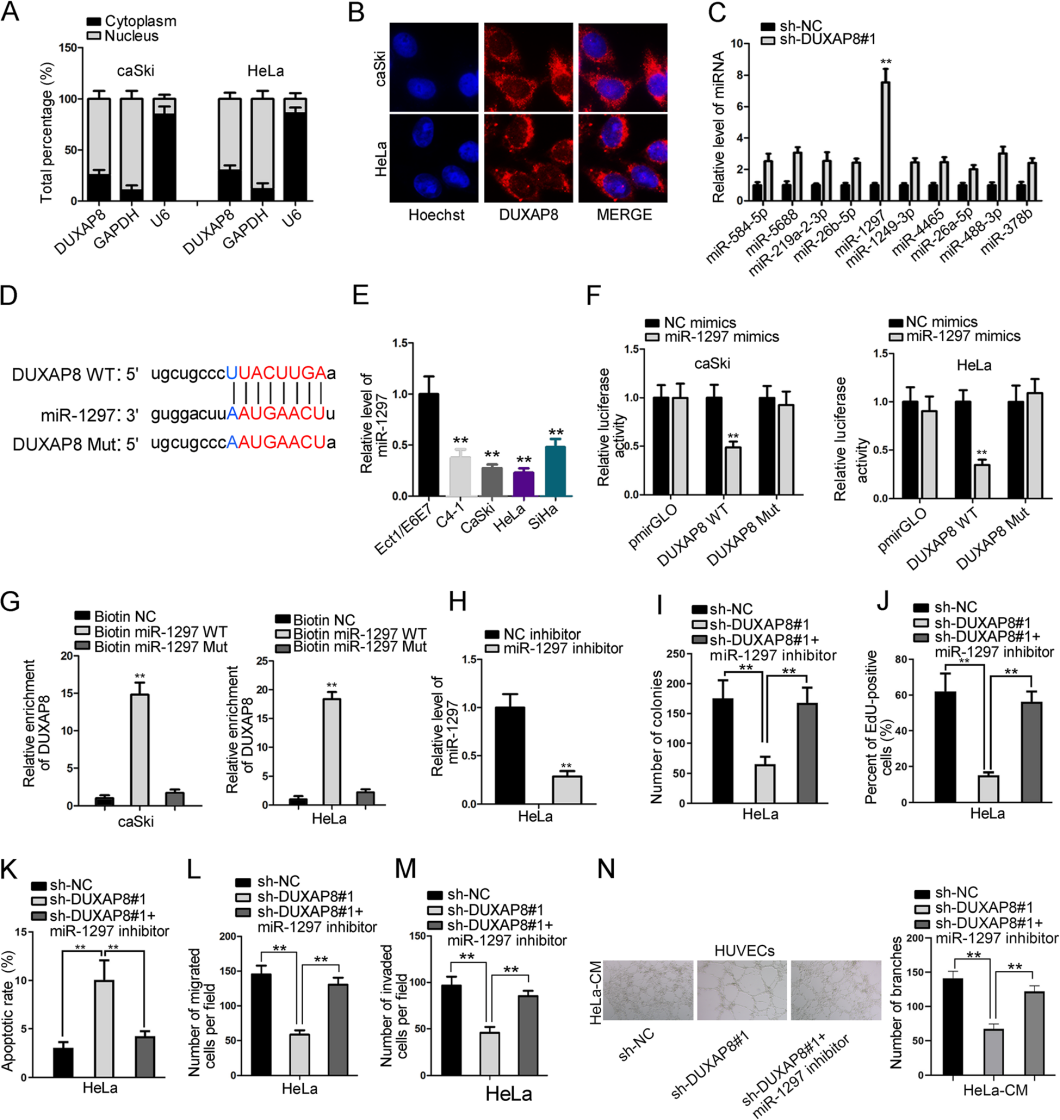
MiR-1297 binds to DUXAP8 and is negatively mediated by DUXAP8. **A-B**. The location of DUXAP8 in caSki and HeLa cells was detected by subcellular fractionation and FISH assays. **C**. RT-qPCR analysis was performed to detect the expression of candidate miRNAs in CC cells with or without DUXAP8 depletion. **D**. Putative and mutant binding sites between DUXAP8 and miR-1297. **E**. The expression of miR-1297 in CC cell line and control cells was determined by RT-qPCR. **F**. Luciferase reporter assays showed that miR-1297 overexpression could impair the luciferase activity of DUXAP8-WT. **G**. RNA pull down showed that DUXAP8 was preferentially enriched by biotin-labeled miR-1297-WT. **H**. The expression of miR-1297 was evaluated by RT-qPCR in two CC cells after transfecting with miR-1297 inhibitor. **I-N**. Rescue experiments were implemented in HeLa cells to elucidate the influence of miR-1297 inhibitor on the cellular activities of DUXAP8-silenced CC cells. **P < 0.01

The ceRNA network is an important posttranscriptional regulatory mechanism of cytoplasmic lncRNAs. From the outcomes of subcellular fractionation and FISH assays, we found that DUXAP8 mainly located in the cytoplasm of caSki and HeLa cells (Fig. 2A, B). This phenomenon indicated the possible ceRNA role of DUXAP8 in CC. By the prediction on starBase (http://starbase.sysu.edu.cn/) database, 10 miRNAs with potential binding capacity to DUXAP8 were detected (Clip data: medium stringency ≥ 2, Class:>7 mer-m8). After performing RT-qPCR analysis, only miR-1297 was found to be conspicuously up-regulated in response to DUXAP8 deficiency (Fig. 2C). Then, we browsed starBase and acquired the putative binding sites between DUXAP8 and miR-1297 (Fig. 2D). Subsequently, miR-1297 was found to be downregulated in CC cells compared with normal ones (Fig. 2E). According to the results of luciferase reporter assays, we found that miR-1297 mimics notably attenuated the luciferase activity of DUXAP8-WT in caSki and HeLa cells, while presented no effects on that of DUXAP8-Mut (Fig. 2F). Moreover, the data of RNA pull down assay manifested that DUXAP8 was enriched only by Biotin miR-1297 WT (Fig. 2G). These results indicated that DUXAP8 directly interacted with miR-1297 and negatively regulated miR-1297 expression in CC cells. We utilized miR-1297 inhibitor to down-regulate the expression of miR-1297 in HeLa cells (Fig. 2H). After conducting a series of rescue experiments, we verified that miR-1297 inhibition distinctively abrogated the cellular effects mediated by silencing DUXAP8 on different behaviors, including cell proliferation (Fig. 2I J), apoptosis (Fig. 2K), migration (Fig. 2L), invasion (Fig. 2M) and angiogenesis (Fig. 2N). To be concluded, miR-1297 was the downstream molecule of DUXAP8 in regulating CC development.

### RCN2 is the potential downstream target of miR-1297

**Fig. 3:**
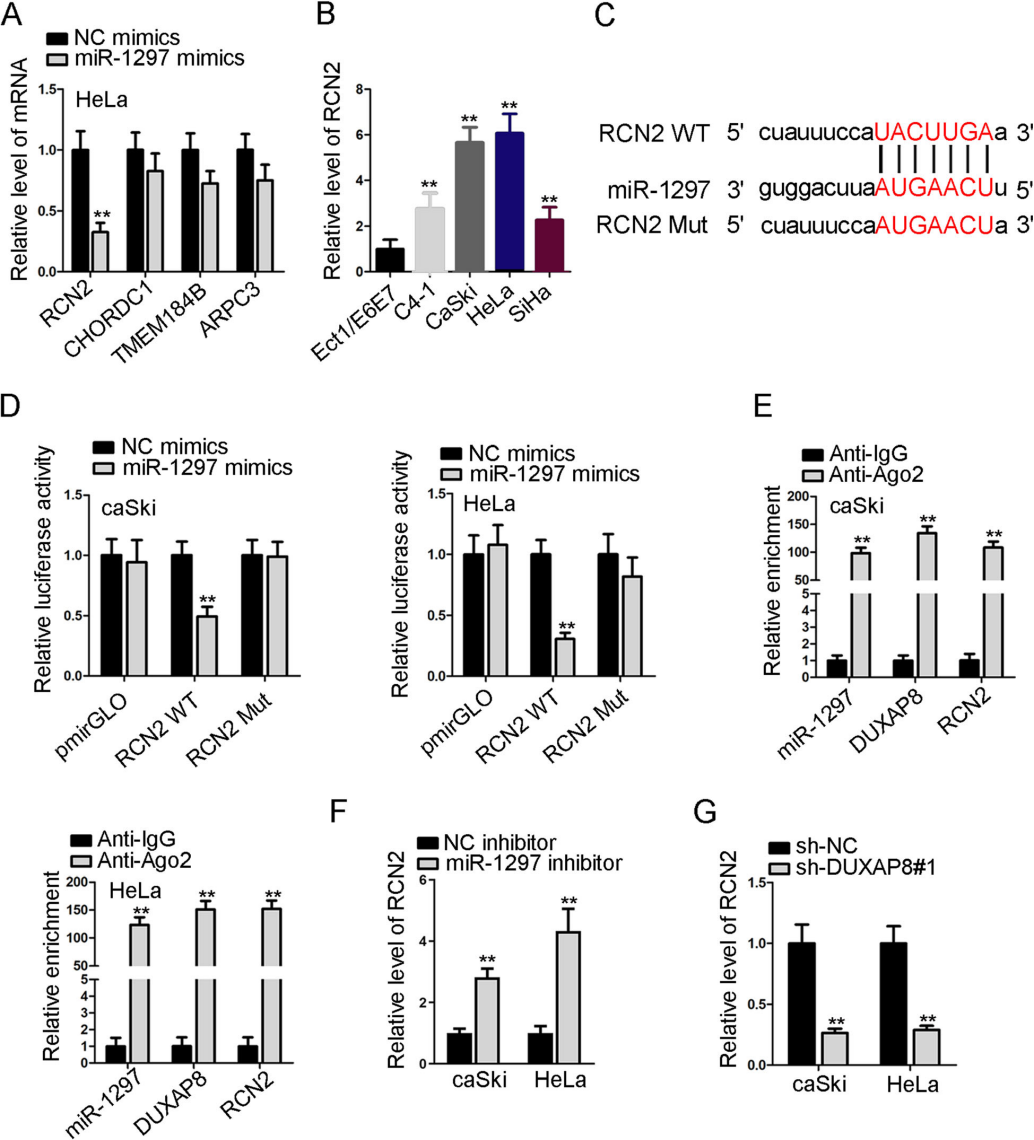
RCN2 is the potential downstream target of miR-1297. **A**. The expression levels of potential target genes for miR-1297 were evaluated by RT-qPCR after overexpressing miR-1297 in HeLa cells. **B**. The expression status of RCN2 in CC cell lines and non-tumor control cells was determined by RT-qPCR. **C**. Putative binding sites between miR-1297 and RCN2 predicted by starBase. **D**. Luciferase reporter assays were performed in caSki and HeLa cells to determine the physical interaction between miR-1297 and RCN2. **E**. RIP assays were performed to study the molecular relationship of DUXAP8, miR-1297 and RCN2. **F-G**. RT-qPCR was performed to study the effects of DUXAP8 suppression on the expression of miR-1297 or RCN2, respectively. **P < 0.01

Numerous studies discovered that lncRNAs could serve as ceRNAs to affect the expression of genes targeted by the shared miRNAs, leading to changes in corresponding biological function in cancer. We used starBase database to search potential targets of miR-1297. As a result, four candidates (RCN2, CHORDC1, TMEM184B and ARPC3) were predicted to interact with miR-1297 under following circumstances (CLIP Data: high stringency ≥ 3; Degradome Data: high stringency ≥ 3, Program Number: 5, Predicted Program: microT, miRanda, miRmap, PITA). Later, RT-qPCR results showed that RCN2 expression was depleted most significantly after HeLa cells was transfected with miR-1297 mimics (Fig. 3A). Also, we observed a significant up-regulation of RCN2 in CC cell lines (Fig. 3B). The putative binding site of miR-1297 in the 3’ UTR sequence of RCN2 was predicted by starBase database (Fig. 3C). Data of luciferase reporter assays demonstrated that miR-1297 overexpression could significantly impair the luciferase activity of RCN2-WT in caSki and HeLa cells, whereas no changes in the luciferase activity was observed in RCN2-Mut group (Fig. 3D). Additionally, RIP assay was used to confirm the potential interaction between miR-1297 and DUXAP8, as well as the binding between miR-1297 and RCN2. It was showed that miR-1297, DUXAP8 and RCN2 were all markedly enriched by antibody targeting Ago2 compared with IgG control group (Fig. 3E). Further, we observed that the expression of miR-1297 was up-regulated by DUXAP8 silence, while that of RCN2 was depleted under DUXAP8 inhibition (Fig. 3F, G). These results supported that DUXAP8 works as a ceRNA of RCN2 in CC by sponging miR-1297.

### DUXAP8 regulates the behaviors of CC cells via up-regulating RCN2 by binding to miR-1297

**Fig. 4:**
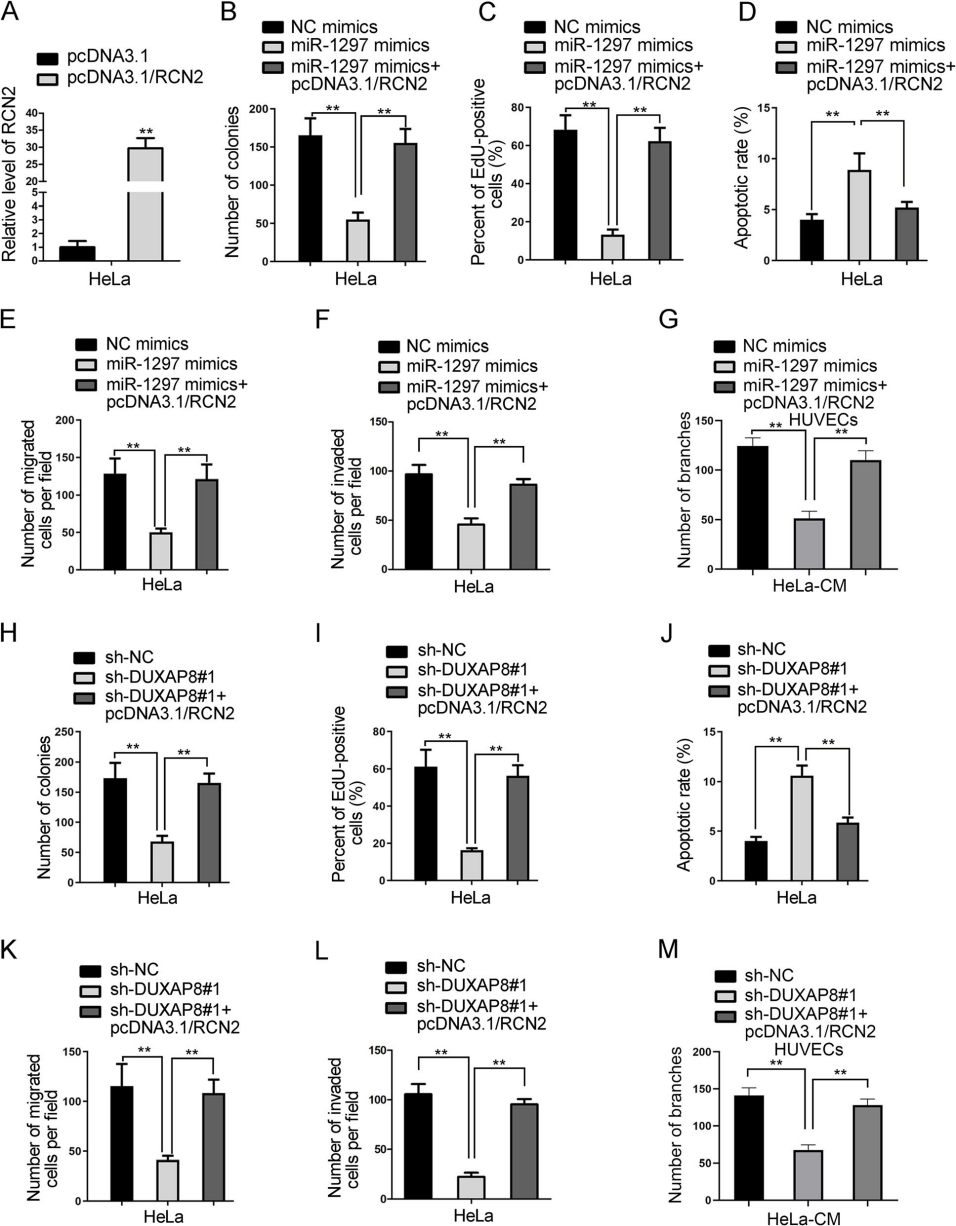
LncRNA DUXAP8 regulates CC cellular behaviors via up-regulating RCN2 by miR-1297. **A**. The expression of RCN2 in HeLa cells after transfecting with pcDNA3.1/RCN2 was detected by RT-qPCR. **B-C**. Colony formation and EdU assays were conducted to explore the proliferation of HeLa cells transfected with NC mimics, miR-1297 mimics or miR-1297 mimics + pcDNA3.1/RCN2. **D**. Flow cytometry analysis was performed to assess the rate of apoptotic cells under different conditions. **E-G**. Transwell and tube formation assays were conducted to determine cell migration, invasion and angiogenesis under diverse contexts. **H-M**. Rescue functional experiments were performed among sh-NC, sh-DUXAP8#1 and sh-DUXAP8#1 + pcDNA3.1/RCN2 groups to explore the effects of RCN2 overexpression on DUXAP8 inhibition-mediated function in HeLa cells. **P < 0.01

To certify whether DUXAP8 regulated CC via serving as a ceRNA, we conducted rescue experiments in HeLa cells. We firstly overexpressed RCN2 in HeLa cells by transfecting with pcDNA3.1/RCN2 (Fig. 4A). It was proved that the proliferation ability of CC cells was suppressed by miR-1297 up-regulation, but restored by RCN2 overexpression (Fig. 4B C). Meanwhile, the apoptotic ability was strengthened by enhanced miR-1297, but weakened by RCN2 overexpression (Fig. 4D). Moreover, the results of transwell assays showed that the inhibitory impacts of miR-1297 elevation on the migration and invasion of HeLa cells were counteracted by RCN2 overexpression (Fig. 4E F). The angiogenic ability restrained by miR-1297 up-regulation was recovered under RCN2 overexpression (Fig. 4G). These findings indicated that the anti-cancer biological function of miR-1297 was mediated by targeting RCN2. Next, we intended to explore whether RCN2 was required for DUXAP8-mediated CC cell function. Based on the results of rescue experiments, we deduced that RCN2 up-regulation could reverse the proliferation-inhibitory impact of silenced DUXAP8 in HeLa cells (Fig. 4H, I). Besides, RCN2 up-regulation could counteract the apoptosis-promoting effect exerted by depleted DUXAP8 (Fig. 4J). Also, the mitigating effects of inhibited DUXAP8 on HeLa cell migration and invasion were abolished in face of RCN2 up-regulation (Fig. 4K, L). The observation from tube formation assay further validated that RCN2 enhancement could recover the suppression of silenced DUXAP8 on angiogenesis (Fig. 4M). Altogether, DUXAP8 aggravates the malignant behaviors of CC cells through boosting RCN2 that is targeted by miR-1297.

### DUXAP8 contributes to in vivo CC tumorigenesis in an RCN2-mediated manner

**Fig. 5:**
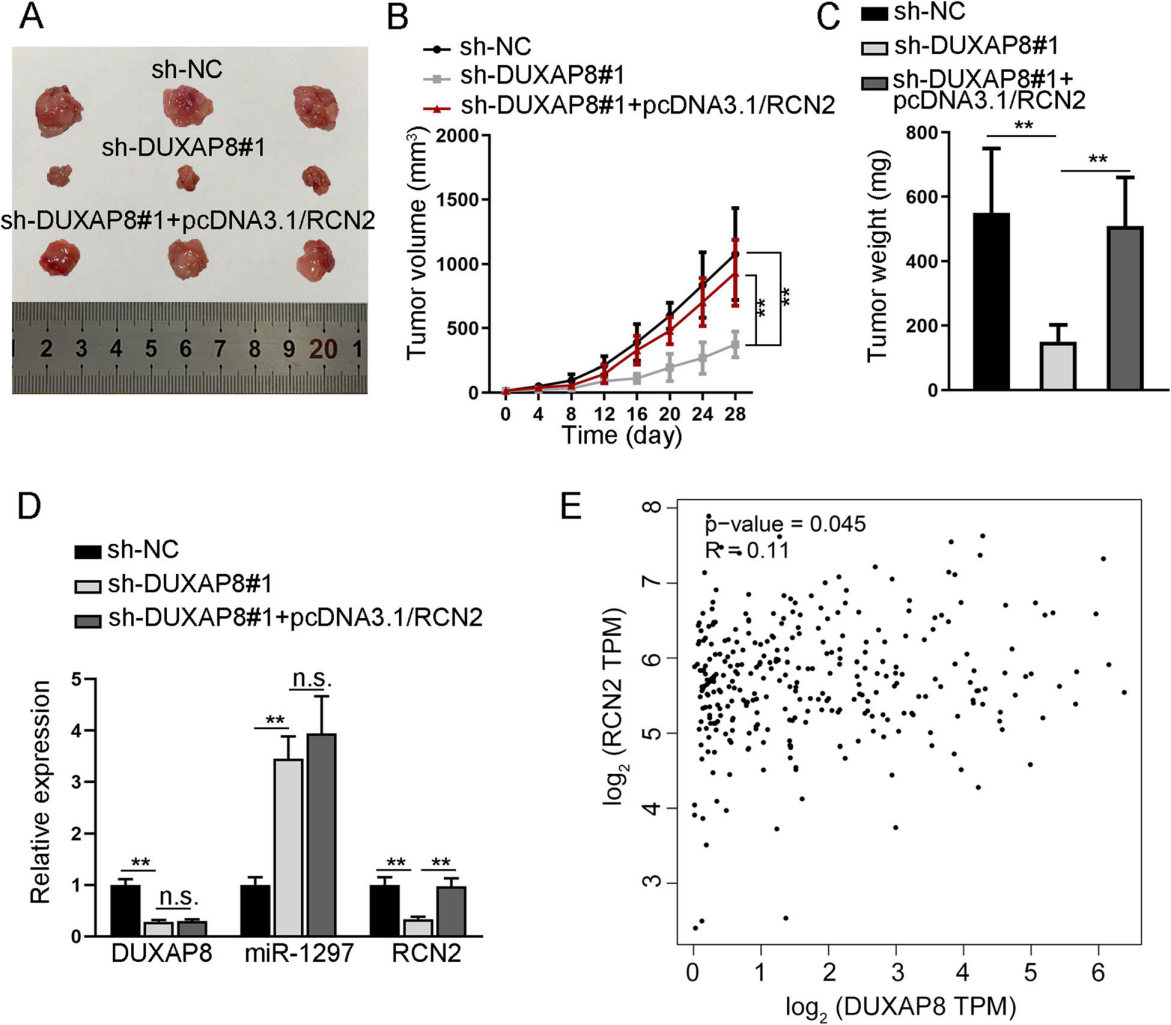
In vivo tumor xenograft growth hindered by suppressed DUXAP8 is recovered under RCN2 overexpression. **A** Representative images of tumors from indicated groups. **B** The growth curve of above tumors recorded during 4 weeks after cell inoculation. **C** The weight of above in vivo tumors from three different groups. **D** The expression of DUXAP8, miR-1297 and RCN2 in these tumors was analyzed via RT-qPCR. **E** The correlation analysis of DUXAP8 and RCN2 in CC analyzed using the TCGA dataset. **P < 0.01, n.s. meant no significance

To further testify the importance of DUXAP8/RCN2 axis in CC tumorigenesis, we implemented in vivo xenograft experiments. According to the final view of xenografts and the corresponding growth curves, we discovered that the growth of tumors derived from DUXAP8-silenced CC cells was evidently obstructed compared to those from the control group, while such obstruction on tumor growth was markedly offset under RCN2 overexpression (Fig. 5A-B). Consistently, changes in tumor weight were similar to that in tumor growth rate in these three groups (Fig. 5C). Moreover, we found that the expression of DUXAP8 was lowered while that of miR-1297 augmented in xenografts from DUXAP8-inhibited cells, while further overexpression of RCN2 had no impacts on these two aspects. Nonetheless, RCN2 level was also declined in tumors with suppressed DUXAP8, which was then recovered upon RCN2 upregulation (Fig. 5D). Besides, we found on TCGA dataset that DUXAP8 is positively correlated with RCN2 in CC tissues (Fig. 5E). Based on these data, we concluded that DUXAP8 facilitated the tumorigenesis of CC by regulating RCN2 that was targeted by miR-1297.

## Discussion

CC is one of the most common diagnosed cancers in female. In gynecologic tumors, the incidence of CC is only preceded by breast cancer [19]. Primary therapies for CC patients includes surgery or a concurrent chemoradiotherapy treatment including cisplatin-based chemotherapy with brachytherapy and external beam radiotherapy [20]. However, the efficacy of cisplatin was compromised due to drug resistance feature of CC cells in treating advanced or recurrent CC [21]. Recently, the anti-cancer research on CC has switched to molecular level [20] while as a novel approach, gene therapy have brought hope to notably improve the survival rate of patients [22]. The underlying regulatory mechanism behind the pathogenesis of CC is worthwhile for anti-cancer research. Extensive studies have revealed that lncRNAs could affect various biological actions, such as transcriptional control, posttranscriptional regulation and the suppression of nuclear transport [23]. Moreover, the important regulatory role of lncRNAs in CC has been disclosed. DUXAP8, as a newly-found lncRNA, has been unveiled to possess oncogenic potential in gastric cancer [24]. Herein, we initially found the aberrant up-regulation of DUXAP8 in CC. Studies have shown that DUXAP8 facilitated cell growth in renal cell carcinoma and served as a promising biomarker in this disease [25]. Loss-of-function assays manifested that DUXAP8 silence suppressed cell proliferation, migration, and invasion in CC. Moreover, silencing DUXAP8 significantly weaken the angiogenesis ability in HUVECs incubated with the CM of CC cells. These observations revealed that DUXAP8 acted as an oncogene in CC, in line with the findings in HCC [26].

Increasing lncRNAs have been found to be involved in the prevalent ceRNA networks, where they could compete with the upstream mediators of mRNAs, miRNAs, for the 3’UTR region of mRNAs, consequently regulating such mRNAs [27, 28]. MiRNAs are a group of endogenous small ncRNAs that can modulate target gene expression at post-transcriptional level and capable of interaction with mRNA-coding genes [29]. Meanwhile, miRNA and lncRNA are key non-coding RNAs because of their negative feedback interaction and regulation on various biological processes by targeting diverse molecular pathways [30]. In our research, miR-1297 was identified as a molecular downstream gene of DUXAP8 from bioinformatics analysis and mechanical experimental exploration. It has been uncovered that miR-1297 exerts inhibitory effects on the development and progression of multiple cancers, such as oral squamous cell carcinoma, [31] pancreatic cancer [32] and gastric cancer [33]. Similarly, miR-1297 has been reported to function as a tumor suppressor to curb cell proliferation and facilitate cell apoptosis in CC [34]. Herein, based on rescue assays conducted in HeLa cells who have a high heterogeneity [35], we discovered that DUXAP8 exerted the oncogenic property in CC via physically binding to miR-1297 and negatively regulating its expression.

Reticulocalbin-2 (RCN2) was validated to elicit an oncogenic function in colorectal cancer [36]. In this study, it was identified as the target gene of miR-1297 by bioinformatics prediction and its level was significantly depleted by miR-1297 overexpression in CC cells. Mechanically, RCN2 overexpression markedly reversed the anti-tumor effects induced by miR-1297 overexpression and DUXAP8 knockdown on CC cells, indicating that DUXAP8 regulated the progression of CC by targeting miR-1297/RCN2 axis. Moreover, data of in vivo experiments further indicated that DUXAP8 contributed to the tumorigenesis of CC via modulating RCN2 that was targeted by miR-1297.

## Conclusions

In sum, we initially found that DUXAP8/miR-1297/RCN2 ceRNA axis elicited important impacts on cell proliferation, migration and invasion and angiogenesis in CC. In other words, DUXAP8 facilitated the malignant progression of CC cells via competitively binding with miR-1297 and upregulating RCN2 both in vitro and in vivo. This result provided new viewpoints into the potential therapeutic effects of DUXAP8 in CC, indicating that DUXAP8 may work as a practical biomarker and a treatment target for CC in the future. Therefore, our findings may shed new lights on the identification of potential novel anti-tumor targets for CC treatment.

## Data Availability

Not applicable.

## Abbreviations

CC: Cervical cancer
ceRNA: Competing endogenous RNA
DUXAP8: Double homeobox A pseudogene 8
EMT: Epithelial mesenchymal transition
lncRNAs: Long non-coding RNAs
ncRNAs: Non-coding RNAs
RT-qPCR: Real-time quantitative PCR
RCN: Reticulocalbin
RCN2: Reticulocalbin-2
RIP: RNA binding protein immunoprecipitation
